# Development of Mathematical Function Control-Based 3D Printed Tablets and Effect on Drug Release

**DOI:** 10.1007/s11095-024-03780-5

**Published:** 2024-10-21

**Authors:** Honghe Wang, Indrajeet Karnik, Prateek Uttreja, Peilun Zhang, Sateesh Kumar Vemula, Michael A. Repka

**Affiliations:** 1 Department of Pharmaceutics and Drug Delivery, School of Pharmacy, The University of Mississippi, University, MS 38677 USA; 2 Pii Center for Pharmaceutical Technology, The University of Mississippi, University, MS 38677 USA

**Keywords:** fused deposition modeling, hot-melt extrusion, prediction modeling, surface equations, three-dimensional printing

## Abstract

**Purpose:**

The application of 3D printing technology in drug delivery is often limited by the challenges of achieving precise control over drug release profiles. The goal of this study was to apply surface equations to construct 3D printed tablet models, adjust the functional parameters to obtain multiple tablet models and to correlate the model parameters with the *in vitro* drug release behavior.

**Methods:**

This study reports the development of 3D-printed tablets using surface geometries controlled by mathematical functions to modulate drug release. Utilizing fused deposition modeling (FDM) coupled with hot-melt extrusion (HME) technology, personalized drug delivery systems were produced using thermoplastic polymers. Different tablet shapes (T1-T5) were produced by varying the depth of the parabolic surface (*b* = 4, 2, 0, -2, -4 mm) to assess the impact of surface curvature on drug dissolution.

**Results:**

The T5 formulation, with the greatest surface curvature, demonstrated the fastest drug release, achieving complete release within 4 h. In contrast, T1 and T2 tablets exhibited a slower release over approximately 6 h. The correlation between surface area and drug release rate was confirmed, supporting the predictions of the Noyes-Whitney equation. Differential Scanning Calorimetry (DSC) and Scanning Electron Microscope (SEM) analyses verified the uniform dispersion of acetaminophen and the consistency of the internal structures, respectively.

**Conclusions:**

The precise control of tablet surface geometry effectively tailored drug release profiles, enhancing patient compliance and treatment efficacy. This novel approach offers significant advancements in personalized medicine by providing a highly reproducible and adaptable platform for optimizing drug delivery.

**Graphical Abstract:**

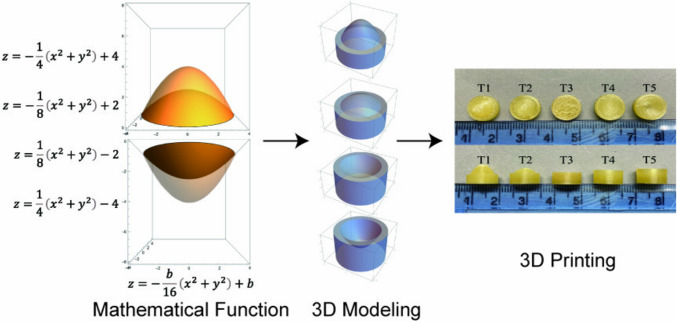

## Introduction

In recent years, 3D printing technology has been widely used to produce various drug delivery systems [1–4]. The most basic and simple shapes, such as cylinders, cuboids, elliptical cylinders, stars, and hearts, are assembled in 3D-printed preparations [5–8]. Further modification of these shapes, for example, partial removal of the center of a cylinder, can produce a shell-shaped tablet to incorporate a core tablet, while flattening or reducing the volume of a cuboid can make a film or implant [2, 9, 10]. These widely used shapes are easy to design and print but have some drawbacks: (1) The changeability of the geometric shapes is small, and the adjustable parameters are few — however, such parameters as curvature and infill patterns significantly impact the physicochemical characteristics of the printed object as the shape and size of the tablet changes [11–13]. (2) the edges of certain printed tablets are sharp, with right-angled cross sections that can easily damage the esophagus. The edges of such printed tablets would have to be filed to make the tablet edible and to increase patient compliance and acceptability [14, 15]. Therefore, there is an urgent need to design and optimize more complex structures based on simple structures.

In mathematical terms, curvature quantifies how much a surface diverges from flat or straight [16]. Curvature can be presented in tablets to ease the swallowing of printed tablets [14]. The change in the shape due to the introduction of curvature also helps to alter the residence time and the disintegration of the tablet [17]. In the case of dosage forms administered via non-oral routes, the curvature of the dosage form aids in introducing the printed dosage form into the body. Caplets and bullet-shaped suppositories have been manufactured multiple times using 3D printing technology [18–21]. Caplet shapes can be designed by filing the edges of a cuboid, and bullet-shaped suppositories also contain curved surfaces. However, the degree of curvature of these formed surfaces is difficult to define and not conducive to production. Similar structural designs lack reproducibility.

In additive manufacturing, surface equations can describe the geometry and shape of the 3D models [22]. These equations are essential for accurately representing and manipulating the surfaces of objects to be printed [23, 24]. Hence, using mathematical functions to describe the surface can make the structure precise, fully controllable, easy to modify, highly reproducible, and even allow for changes in the curvature of the surface to control the drug release rate.

Fused deposition modeling (FDM) is an additive manufacturing technology used to manufacture personalized drug delivery systems [25]. It usually involves using Hot-melt extrusion (HME) technology to produce filaments suitable for printing [26]. The coupling of FDM and HME technologies makes for a versatile platform to fabricate dosage forms using thermoplastic polymers, which are also suitable for fitting the administration of drug substances [27–29]. The HME process is a widely researched technique for manufacturing amorphous solid dispersion (ASD) systems [30, 31]. In HME, mixing and extruding the API with a hydrophilic and thermoplastic polymer matrix at elevated temperatures enhances the drugs’ solubility and bioavailability [32–34]. The HME filaments can then be fed into the FDM printer and melted and extruded through a nozzle [27]. The nozzle moves over a printing bed and constructs a layer-by-layer drug delivery system. The printing process is directed according to the Standard Tessellation Language (.stl; STL) file, which describes the surface geometry of the 3D object generated using computer-aided design (CAD) software. The design employs a mesh of triangles without any color, texture, or other attributes to illustrate the surface morphology of the object. A slicing software converts the STL file into horizontal layers and generates toolpaths for the 3D printer to follow to deposit the material [35]. The output is a G-Code file, which is understood by the 3D printer, thereby controlling the printer's movements, nozzle temperature, and other parameters [36]. Variations in the shape of the 3D-printed tablets significantly affect the critical quality attributes of the tablets, including release characteristics of the drug from the tablet [37]. Thus, dimensional precision is a necessity to ensure the reproducibility and reliability of the dosage forms [38].

Based on the above advantages of 3D printing, mathematical surface equations, and the lack of precision in the process affecting the dosage form attributes, this work aimed to (1) apply surface equations to construct 3D printed tablet models and to produce water-soluble polymer (polyvinyl alcohol, PVA) based 3D printed tablets using hot melt extrusion and FDM 3D printing techniques, and (2) adjust the functional parameters to obtain multiple tablet models and to correlate the model parameters with the *in vitro* drug release behavior.

## Materials and Methods

### Materials

Acetaminophen (APAP) was purchased from SpecGx LLC (Raleigh, NC, USA). Parteck® MXP PVA 3–82 (Polyvinyl alcohol, MWT: 32 kDa) was donated by EMD Millipore (Burlington, MA, USA). All other reagents were of analytical grade.

### Methods

#### Formulation

Acetaminophen (APAP), a high-solubility drug, was used as a model drug. The formulation was composed of 15% (w/w) of APAP and 85% (w/w) of PVA.

#### Hot-Melt Extrusion

In this study, physical mixtures of formulation ingredients were extruded using a co-rotating, 40:1 length-to-diameter ratio twin-screw extruder (Thermo Fisher Scientific, Waltham, MA, USA) with screws that had an 11 mm diameter. Eight electrically heated zones were adjusted to 160 °C, and the extruder was run at a screw speed of 50 rpm. A 1.5 mm round die, and a conventional screw design with three mixing zones supplied by Thermo Fisher Scientific were used to extrude filaments appropriate for 3D printing. Before being fed into the 3D printer, the extruded filaments were straightened and cooled using a conveyor belt at the extrusion speed.

#### 3D Design of Tablets

**Fig. 1:**
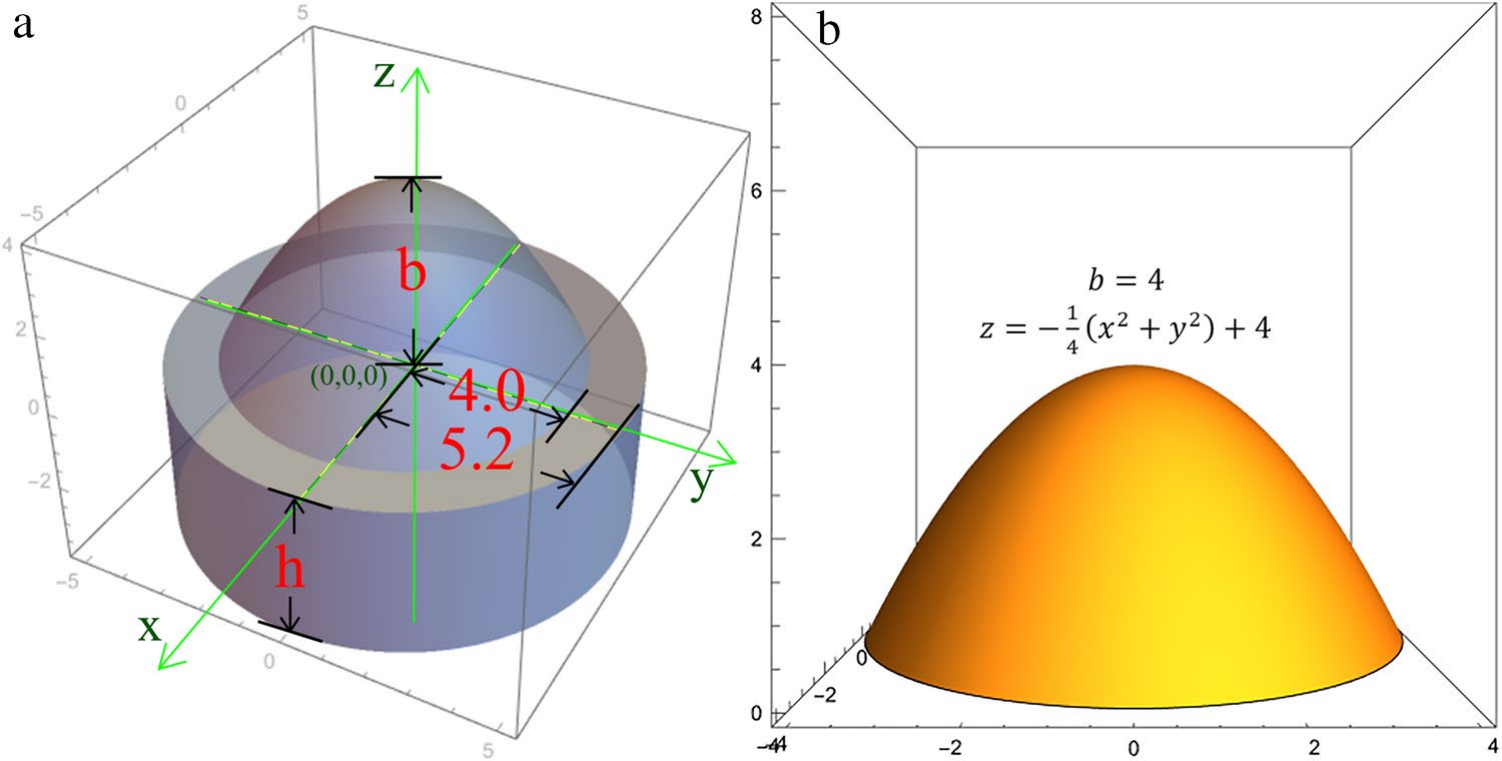
Dimensions of 3D printed tablets. (**a**) the view of tablet design (units: mm). (**b**) paraboloid equation.

First, a cylinder with a radius of 5.2 mm and a height of 5 mm (*h*_*0*_) was designed. A Cartesian coordinate system for a three-dimensional space was established on the top center of the cylinder, with the top center being the origin (Fig. 1a). Deformations were made upwards or downwards within a circle with a radius of 4 mm on the *XOY* plane. The protrusion depth was set to *b*, and when *b* was positive, the tablet showed an upward protrusion. In contrast, when *b* was negative, the tablet showed a downward depression (Fig. 1b). The shape of the protrusion was described using the parabolic equation in this space.

1 $$z=-\frac{b}{16}\left({x}^{2}+{y}^{2}\right)+b$$

When *b* > 0, the parabolic surface is a concave paraboloid, and when *b* < 0, the parabolic surface is a convex paraboloid. Keeping the total volume of the tablet constant (i.e., the volume of the initial cylinder), when the depth *b* of the paraboloid changes, the height of the cylinder in the *z* < 0 area changes accordingly.

The volume *Vc* enclosed by the paraboloid and the *XOY* plane is:

2 $${V}_{c}=\underset{V}{\iiint }dxdydz$$

Substituting the parabolic Eq. (1) into the Eq. (2) and performing the triple integral, we get:

3 $${V}_{c}=4{\int }_{0}^{4}dx{\int }_{0}^{\sqrt{{4}^{2}-{x}^{2}}}dy{\int }_{0}^{-\frac{b}{16}\left({x}^{2}+{y}^{2}\right)+b}dz=8b\pi$$

Next, the volume *Vc* and the part of the cylinder body follow the following equation:

4 $$\pi {r}^{2}{h}_{0}-{V}_{c}=\pi {r}^{2}h$$

Then, substituting the parameters of the paraboloid and the cylinder into the equation, we get:

5 $$h={h}_{0}-\frac{8b}{{r}^{2}}=5-\frac{50b}{169}$$

**Fig. 2:**
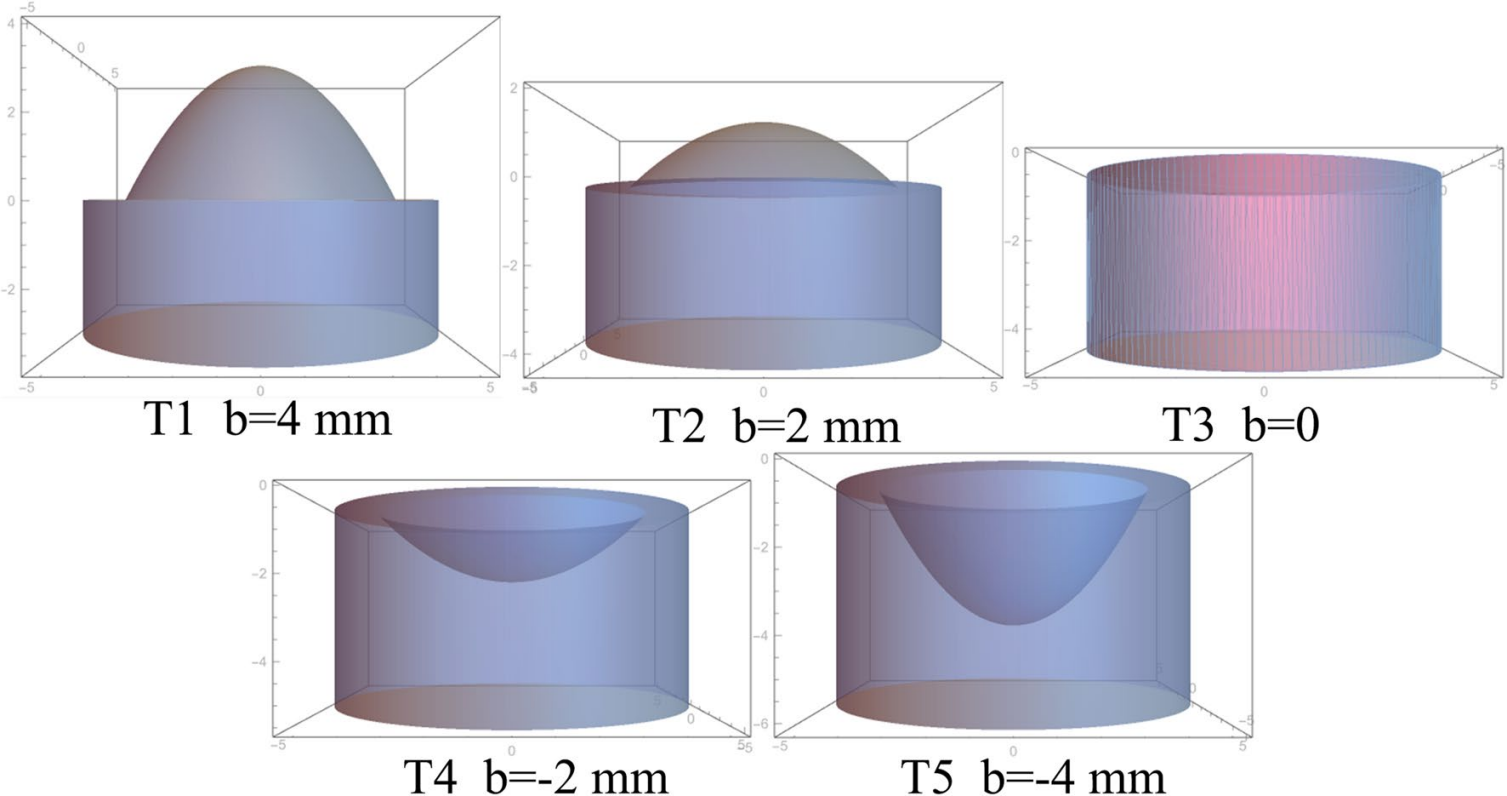
Dimensions of 3D printed tablets.

In this experiment, *b* values of 4, 2, 0, -2, and -4 mm were chosen, and five different curved paraboloids were obtained, which were used to construct five different tablet shapes: T1, T2, T3, T4, and T5 (Fig. 2).

At last, the ContourPlot3D function of the paraboloid was performed using the mathematical software Wolfram Mathematica 13.1 (Wolfram Research, Inc., Champaign, IL, USA), with PlotPoints set to 40 to generate a 3D contour and then exported to a STL file. The STL file of the paraboloid was imported into the 3D design software Blender (Blender Foundation, Amsterdam, North Holland, Netherlands), and the remaining main part of the cylinder was modeled based on the calculated *h* value from Eq. (5). Finally, the complete tablet 3D structure was exported as an STL file for slicing and printing.

#### Fused Deposition Modeling 3D Printing

A commercial FDM Prusa i3 MK3 3D printer (Prusa Research, Prague, Czech Republic) with a 0.4 mm nozzle was utilized to print the tablets, and slicing software Ultimaker Cura 5.3.0 (Ultimaker B.V., Utrecht, Utrecht, Netherlands) was used to do the slicing and printing. The printing parameters are as follows: layer height, 0.15 mm; infill pattern, lines; one wall line with a 0.4 mm wall thickness; no extra top/bottom layers; no build plate adhesion was used. The printing temperature was 180 °C, while the build plate temperature was 50 °C, and the tablets were printed at 100% infill density at a speed of 50 mm/sec.

#### Differential Scanning Calorimetry (DSC)

A differential scanning calorimetry (DSC) system (TA Instruments, New Castle, DE, USA) was employed to assess the thermal stability and crystallinity of the samples. Acetaminophen (APAP), polyvinyl alcohol (PVA), the physical mixture of these ingredients, and the 3D-printed tablets were tested in this investigation. Samples weighing 5–8 mg were sealed within Tzero aluminum pans, set up on the testing platform, and heated from 25°C to 250°C at 10°C/min. Nitrogen was used as the purge gas at a 50 mL/min flow rate. The data were recorded, and the DSC thermogram was produced using the associated Trios analyzing software.

#### Fourier Transform Infrared Spectroscopy (FTIR)

An Agilent Cary 630 ATR Fourier transform infrared spectroscopy (FTIR) spectrophotometer (Agilent Technologies, Santa Clara, CA, USA) was employed to characterize molecular structures and explore functional group interactions. Spectra were collected across a wavenumber range of 600 – 4000 cm^−1^ with a resolution of 4 cm^−1^. The FTIR spectra of the pure APAP, polyvinyl alcohol, and milled printed tablets were acquired for analysis.

#### Scanning Electron Microscope (SEM)

The surface morphology of the cross-sections of the printed tablet was analyzed utilizing a JSM-7200FLV Scanning Electron Microscope (JEOL, Peabody, MA, USA) operating at an accelerating voltage of 5.0 kV. Before imaging, all samples were affixed to SEM stubs using double-adhesive tape. Subsequently, the samples underwent sputter-coating with Gold and Palladium in a ratio of 60:40 under an argon atmosphere, facilitated by a fully automated Denton Desk V TSC Sputter Coater (Denton Vacuum, Moorestown, NJ, USA). Samples were observed at a magnification of fifty times.

#### 3-Point Bend Test

The three-point test can be employed to assess filaments’ printability, ensuring the printing process’s feasibility. In this study, the three-point test followed the Repka-Zhang method [27]. A TA-XT2 analyzer (Texture Technologies, Hamilton, MA, USA) equipped with a TA-92N probe set and a 5 kg load cell capacity was utilized for the flexibility and brittleness assessments. The filament samples were cut into 6 cm rods and positioned on a 3-point bend rig with a 25 mm width gap on the sample fixture. The knife-shaped probe descended until it reached 20 mm below the initial position of the tested sample. Pre-test, test, and post-test speeds were all set to 10 mm/sec, with a trigger force of 5.0 g. Each filament was subjected to testing six times.

#### *In vitro* drug release

The *in vitro* drug release from the 3D printed tablets was assessed using a United States Pharmacopeia (USP) dissolution apparatus II (Hanson SR8-plus, Hanson Research, CA, USA) with the temperature maintained at 37.0 ± 0.5℃ and a paddle rotation speed set to 50 rpm. A pH 6.8 phosphate buffer was used as dissolution medium. Each formulation was tested in triplicate, with samples collected at 10, 20, 30, 45, 60, 90, 120, 180, 240, 300, 360, and 480 min for analysis. The released acetaminophen was measured using high-performance liquid chromatography (HPLC) equipment (Waters Corp. Milford, MA, USA), fitted with a SymmetryShieldTM RP18 C18 column (250 mm × 4.6 mm, 5.0 μm) and set to a detection wavelength of 243 nm. Data was analyzed using Empower software (v. 2, Waters Corp.). A sample injection volume of 10 μL was used, with an isocratic flow rate of 1.0 ml/min. The mobile phase had a methanol-to-water ratio of 70:30 (v/v), while the column temperature was set at 25°C. The retention time of APAP was approximately 3.3 min, with a total run time of 7 min.

#### Drug Release Kinetics

The *in vitro* drug release data of T1, T2, T3, T4, and T5 tablets were fitted into zero-order, first-order, the Hixson-Crowell equation, and the Ritger-Peppas equation to assess the drug release mechanisms (Table I) [39, 40].

**Table I:** Drug release kinetics equations

Zero-order	First-order:	Hixson-Crowell equation	Ritger-Peppas equation
$\frac{Q}{{Q}_{\infty }}=kt$	$\frac{Q}{{Q}_{\infty }}=1-{e}^{-kt}$	${\left(1-\frac{Q}{{Q}_{\infty }}\right)}^\frac{1}{3}=1-kt$	$\frac{Q}{{Q}_{\infty }}=k{t}^{n}$

Where *Q/Q*_*∞*_ is the cumulative percentage of drug release at time *t*. *k* is the constant determined by fitting each formula. *n* is the exponent of the Ritger-Peppas formula

## Results and Discussion

### Formulation

According to the basic dissolution theory of the Noyes-Whitney equation, the surface area is an important factor that affects dissolution behavior [41]. In this experiment, the volume of tablets was a constant value, and the surface area of tablets was manipulated by adjusting the depth of the paraboloid. In order to obtain a one-to-one mapping between the surface area and depth, the tablet surface area was calculated accurately.

The surface area of the paraboloid *Ac* was calculated using the formula for calculating spatial surface area:

6 $${A}_{c}=\underset{D}{\iint }\sqrt{1+{\left(\frac{\partial z}{\partial x}\right)}^{2}+{\left(\frac{\partial z}{\partial y}\right)}^{2}}dx dy$$

The paraboloid was projected onto the *XOY* plane to obtain a circular region, and a double integral operation was performed within the circular region:

7 $${A}_{c}=4{\int }_{0}^{\pi /2}d\theta {\int }_{0}^{4}\sqrt{1+\frac{{b}^{2}}{64}{\rho }^{2}}\rho d\rho =\frac{16\pi (-8+{(4+{b}^{2})}^{3/2})}{3{b}^{2}}$$

The surface area of the entire tablet was then calculated using the formula:

8 $$A={A}_{c}+ 2\pi {r}^{2}-\pi {{r}_{0}}^{2}+2\pi rh$$

Substituting the parameters of the cylinder and the surface area of the paraboloid *Ac*, the following equation was obtained:

9 $$A=(\frac{952}{25}+\frac{52}{5}(5-\frac{50b}{169})+\frac{16(-8+{(4+{b}^{2})}^{3/2})}{3{b}^{2}})\pi$$

When *b* was set to 4, 2, 0, -2, and -4 mm, the theoretical surface area *A* of the tablet was 329.62, 324.93, 333.26, 363.60, and 406.94 mm^2^, respectively. It is worth noting that the limit value of the *A* function as *b* approaches 0 is entirely consistent with the surface area value of the original undeformed cylinder. Therefore, the tablet surface area is continuous when *b* = 0.

**Fig. 3:**
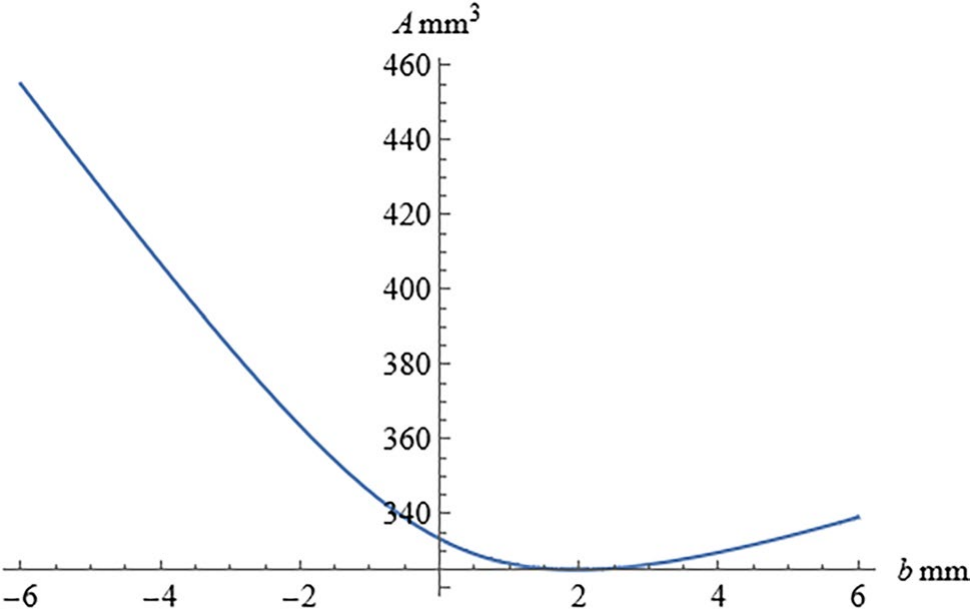
The relationship between the tablet surface area and the paraboloid depth.

**Fig. 4:**
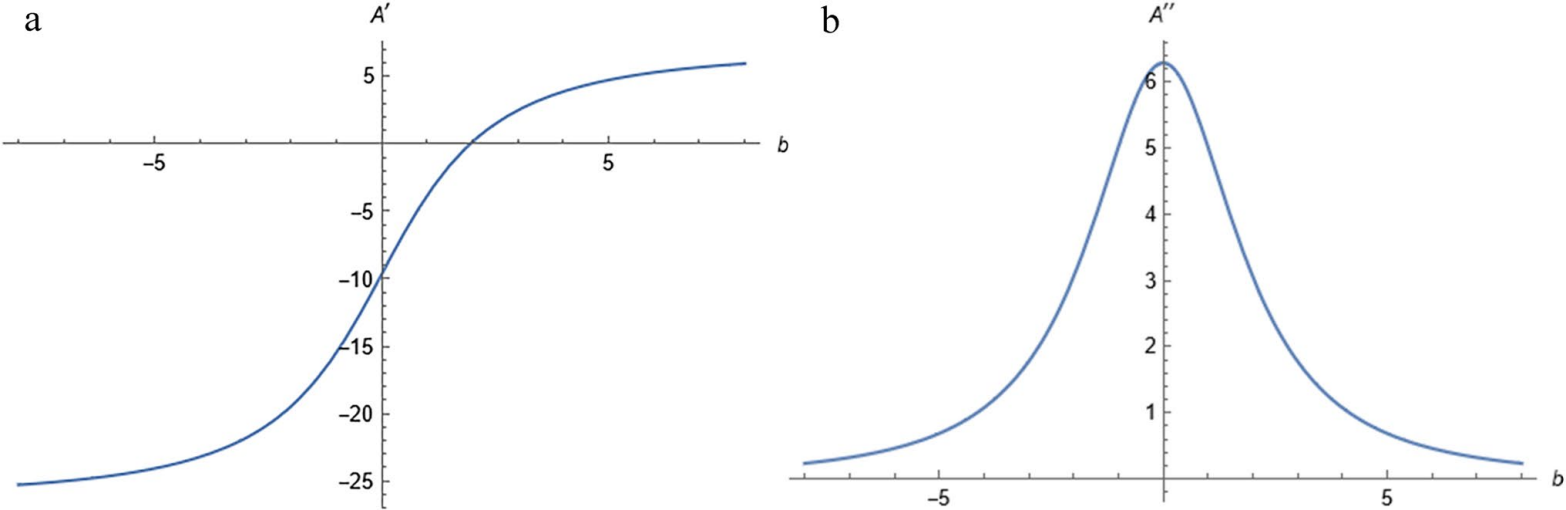
The derivative function curves of the tablet surface area versus the paraboloid depth. (**a**) first derivative. (**b**) second derivative.

As shown in the Fig. 3, when *b* < 0, the surface area of the tablet gradually decreases as *b* increases. When *b* > 0, A first decreases and then increases as *b* increases. When *b* = 1.95mm, *A* is the minimum value of 324.929 mm^2^, and the surface area changing rate is the smallest. From the first and second derivative graphs, the surface area changing rate increases most rapidly as *b* approaches 0 (Fig. 4).

The first and second derivatives of the surface area function *A* were calculated as follows:

10 $$A'=\frac{dA}{db}=(-\frac{40}{13}+\frac{16\sqrt{4+{b}^{2}}}{b}-\frac{32(-8+{(4+{b}^{2})}^{3/2})}{3{b}^{3}})\pi$$

11 $$A''=\frac{{d}^{2}A}{{db}^{2}}=(\frac{16}{\sqrt{4+{b}^{2}}}-\frac{48\sqrt{4+{b}^{2}}}{{b}^{2}}+\frac{32(-8+{(4+{b}^{2})}^{3/2})}{{b}^{4}})\pi$$

The surface defined by the function is theoretically smooth without edges or line segments. However, while importing the surface into 3D design software, the surface will be reconstructed by vertices, edges, faces, and polygons, causing volume and surface area deviations. The surface area and volume of tablets with five different specifications were calculated using the 3D printing plugin in Blender, and their percent deviations were calculated. Table II shows that the percent deviations are all less than 0.06%. Therefore, designing the tablet structure using the function is feasible and can be correlated with dissolution behaviors.

**Table II:** Theoretical mathematical values and 3D software calculation values of tablet dimensions

		T1	T2	T3	T4	T5
Theoretical mathematical value	*b* (mm)	4	2	0	-2	-4
	*h* (mm)	3.817	4.408	5.000	5.592	6.183
	*A* (mm^2^)	329.615	324.933	333.260	363.599	406.947
3D software calculation value	Surface area (mm^2^)	329.675	325.081	333.199	363.714	406.957
	Percent deviation	0.0182%	0.0455%	-0.0183%	0.0316%	0.0025%
	Volume (mm^3^)	424.822	424.953	424.619	424.995	424.815
	Percent deviation	0.0186%	0.0494%	-0.0292%	0.0593%	0.0170%
Real value	Weight (mg) (mean ± SD, n = 6)	529.7 ± 3.87	525.6 ± 3.38	524.2 ± 3.50	530.7 ± 6.42	526.2 ± 3.37

**Fig. 5:**
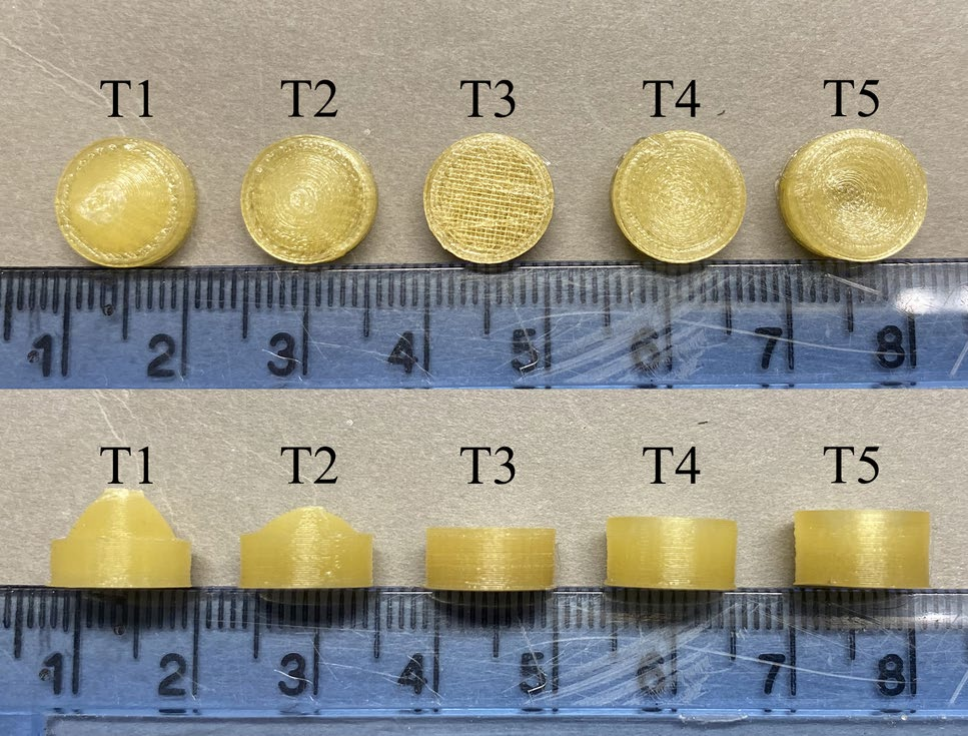
The top and side views of 5 types of 3D printed tablets.

All printed tablets were manufactured with 100% infill density, resulting in a relatively slow dissolution rate compared to tablets with lower infill density. Polyvinyl alcohol (PVA) has excellent water solubility, and the filaments produced from this formulation exhibit sufficient hardness to meet printing requirements. The aim was to finish the dissolution process within a few hours instead of over 20 h; hence, PVA was chosen as the excipient matrix material. The printed tablets appeared transparent yellowish, with distinct layers observed upon side view (Fig. 5). The cylindrical parts displayed uniformity in size above and below, with no evidence of layer separation, high-thermal expansion, or color variation. The surface of the filament was smooth, extruded smoothly during printing, and did not cause any blockages in the printer gears or breakages beneath the gears. Additionally, the filament did not experience abrasion, tear, or residue accumulation due to gear compression, indicating it is a suitable material for printing.

### Differential Scanning Calorimetry

**Fig. 6:**
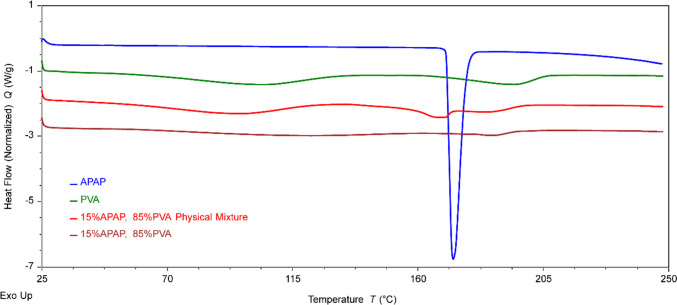
DSC thermogram of acetaminophen, PVA, physical mixture and printed tablets.

DSC is a prevalent solid-state characterization method for identifying thermal transitions in polymeric materials. It measures the heat necessary for phase transitions. In the DSC thermogram of APAP (shown in Fig. 6), an endothermic melting peak emerged around 172°C. The physical mixture showed a tiny endothermic peak of APAP, indicating a portion of APAP was dissolved in molten PVA polymer during the DSC measurement. The endothermic melting peak was notably absent in the printed tablets, suggesting the dispersion of APAP within the polymer.

### Fourier Transform Infrared Spectroscopy

**Fig. 7:**
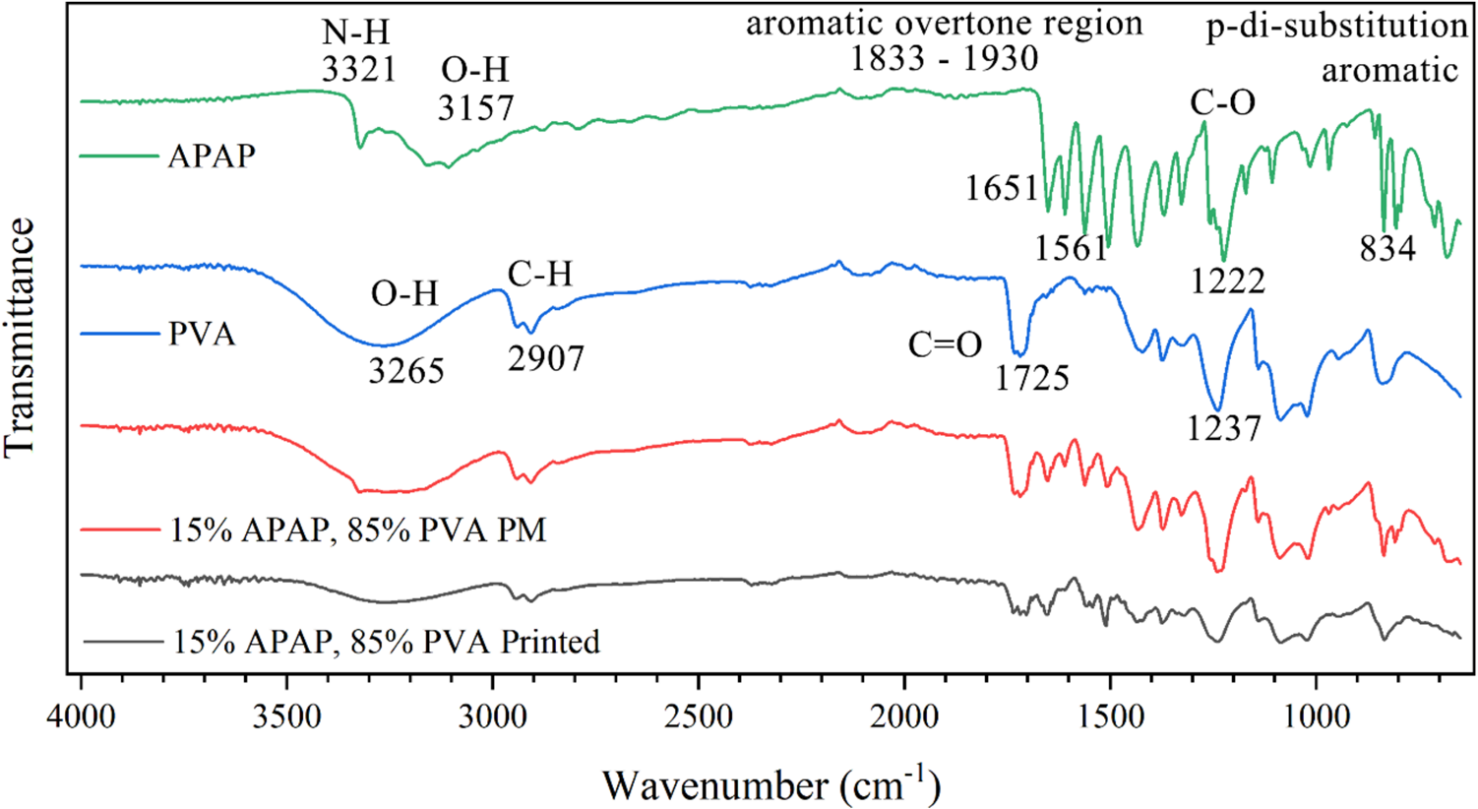
FTIR spectra of acetaminophen, PVA, physical mixture, and printed tablets.

ATR-FTIR was employed to explore potential molecular interactions between APAP and PVA. The spectra of pure APAP, PVA, physical mixture, and printed tablets were obtained (Fig. 7). In the APAP spectra, the broadband at 3157 cm^−1^ corresponds to the phenolic O–H stretching vibrations. At the same time, the sharp band at 3321 cm^−1^ indicates N–H amide stretching vibrations. The band at 1833—1930cm^−1^ corresponds to the aromatic overtone region. The band at 1505 cm^−1^ is related to aromatic C = C stretch stretching vibrations. The band at 1651 cm^−1^ corresponds to the C = O stretching in the amide group. The band at 1561 cm^−1^ represents N–H amide bending, and the band at 1222 cm^−1^ indicates the C-O stretching vibrations. The appearance of the band at 834 cm^−1^ aligns with p-di-substitution aromatic C–H bending. In the PVA spectrum, the band at 1725 cm^−1^, and 1237 cm^−1^ correspond to the C = O stretching and the C-O stretching in the ester groups, respectively [42, 43]. The band at 3265 cm^−1^ is attributed to O–H stretching vibrations, while the band at 2907 cm^−1^ indicates the C-H stretching vibrations. Notably, no discernible bands emerged or disappeared in the spectra of the physical mixture and the printed tablet. This observation suggests that APAP and PVA did not undergo chemical reactions during printing. Furthermore, there were no evident shifts observed in hydrogen bond acceptor or donor groups, thus indicating the absence of hydrogen bonding between the active pharmaceutical ingredient (API) and the excipient.

### Scanning Electron Microscope

**Fig. 8:**
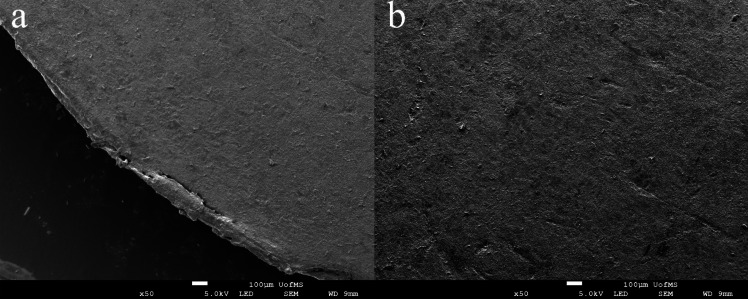
SEM images of the cross-section of the printed tablet at × 50 magnification level. (**a**) the edge of the tablet sample. (**b**) the center of the tablet sample.

The SEM samples for observation were obtained by cutting a complete printed tablet T1 along the layer direction using a razor blade. As shown in Fig. 8, the cross-section of the cut was smooth and intact, without the presence of large individual particles or stringing commonly associated with the printing process. The printing parameter "line width" was set to the diameter of the printer nozzle, which is 0.4 mm. No huge gaps between lines were observed in Fig. 8. However, slight traces were visible in Fig. 8b, which indicates that employing a 100% infill density can result in the printing of tablets with relatively compact and uniform internal structures. In the context of 3D printed dosage forms, the outer walls generally have a 100% printing density, and parameters can be adjusted to modify the number and thickness of these walls. The outer walls determine the shape of the printed object.

On the other hand, the infill density of the internal structure is adjustable, and reducing the infill percentage to increase the porosity of the internal space is a common strategy to accelerate drug dissolution rates [44]. In this experiment, an infill density of 100% was chosen for the internal structure, consistent with the outer wall density, to minimize internal porosity. This approach helps prevent water infiltration along large gaps during the dissolution process, ensuring that the external surface area of the tablet remains in direct contact with water rather than the internal structure. Furthermore, a dense structure also minimizes differences in weight among tablets of five different shapes.

### 3-Point Bend Test

**Fig. 9:**
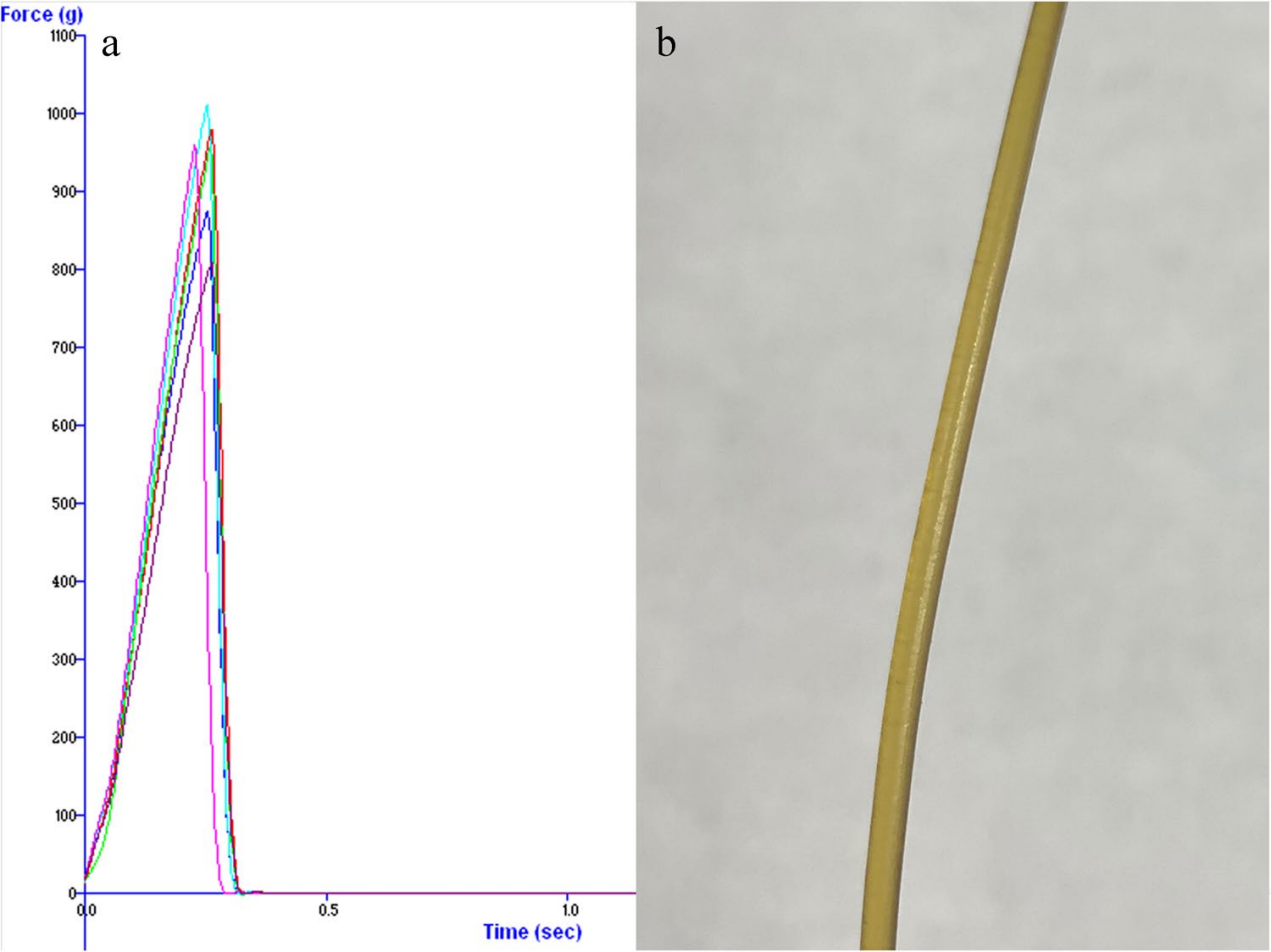
(**a**) The force–time curve of the 3-point bend test of filament, (**b**) The prepared filament for FDM 3D printing.

The filament appeared pale yellow, with a smooth surface, and no powder deposition was visible to the naked eye (Fig. 9). The filament printability is affected by both softness and brittleness. Brittle filaments are prone to fragmentation when subjected to the gears inside the 3D printer, making it challenging to feed them into the printer [45]. Conversely, soft filaments are difficult to load into the printer, exhibit low abrasion resistance, and often cause blockages at the print head, as they cannot be conveyed effectively by gears [27].

The 3-point bend test assesses a filament’s breaking force, breaking distance, and breaking stress. Breaking stress is calculated by dividing the breaking force by the cross-sectional area of the filament. In the 3-point bend test graph, the initial straight line represents the elastic region. Filaments exhibit elasticity only within the elastic region, meaning they regain their original shape upon force removal. Hooke’s law was employed to evaluate filament printability. It has been proven that Hooke’s law ($F=-kx$) can be used to evaluate the printability of filaments [36]. According to Hooke’s law, $F=-kx$, where “*F*” represents stress when the filament's center is displaced by distance “–*x*.” In order to ensure data accuracy along the straight elastic curve, a 2-mm bending distance was selected. Filaments with higher “*k*” values demonstrate improved printability. With a "*k*" value significantly greater than 40 g/mm^3^, the filament exhibited excellent printability [36]. This high success rate in printing enhanced reliability and reduced weight difference among printed tablets (Table III).

**Table III:** The 3-Point Bend Test of Filaments (Mean ± SD, n = 6)

Force (g)	Distance (mm)	Force at 2 mm (g)	Stress at 2 mm (g/mm^2^)	*k* (g/mm^3^)
932.8 ± 75.2	2.56 ± 0.13	749.75 ± 71.18	330.32 ± 31.36	165.16 ± 15.68

### *In Vitro* Drug Release Study

**Fig. 10:**
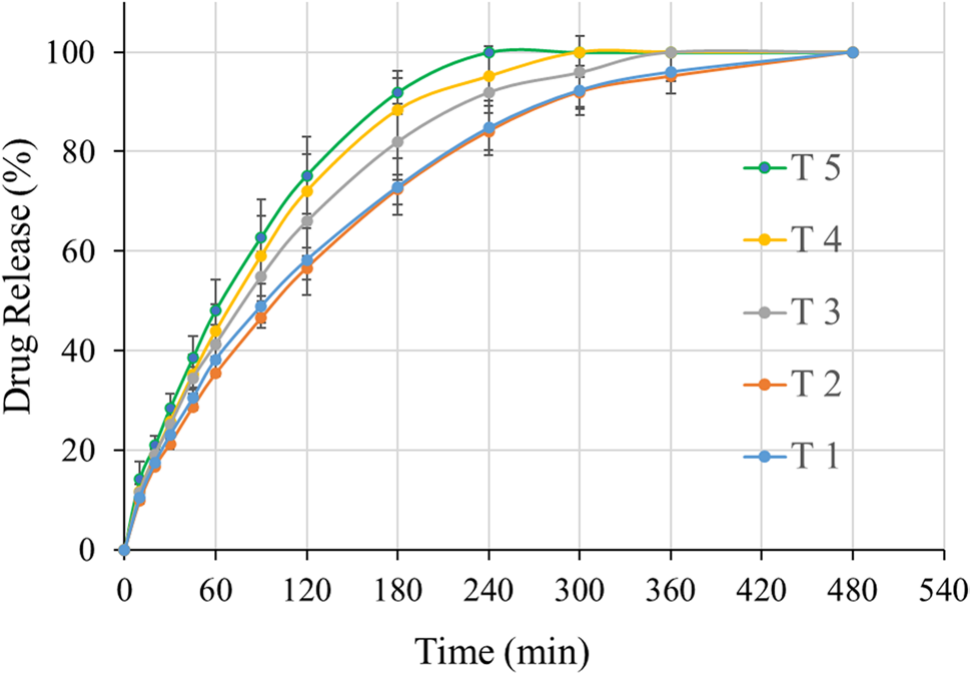
The dissolution profiles of 3D printed tablets.

The dissolution profiles of five different-shaped tablets are shown in Fig. 10. Tablet T5 exhibited the fastest dissolution rate, with drug release completed within 4 h. Tablets T1 and T2 dissolved at a slower and similar rate, with complete drug release taking approximately 6 h. Tablet T5 had the greatest concavity and, thus, the largest surface area, followed by T4 and T3. Tablet T2, with a “*b*” of 2 mm, was very close to the theoretical minimum point *b* = 1.95 mm, resulting in the slowest dissolution rate. According to the Noyes-Whitney equation, the drug release rate is directly proportional to the surface area of the dosage form. These experimental results align with this theory.

### Drug Release Kinetics

In this study, the 3D-printed tablets were composed of a single composition, and only one formulation was used to print the entire tablet without additional shells or layers by other formulations. PVA, a water-soluble matrix, was used, resulting in tablets gradually decreasing size during dissolution. Therefore, the release kinetics formulas mentioned above are all applicable. The fitting results are presented in Table IV, obtained by fitting the drug release percentage (%) versus time (*h*) curves with these formulas. From the *R*^*2*^ values, compared to the zero-order model, the first-order, Hixson-Crowell, and Ritger-Peppas models exhibited more precise fittings (*R*^*2*^ greater than 0.99). For the Ritger-Peppas model, when 0.45 < *n* < 0.89, the kinetics are characterized as anomalous (non-Fickian) transport [39]. In this experiment, the exponent "*n*" for the five-tablet dissolution curves ranged from 0.65 to 0.75. Therefore, the release behavior of the tablets conforms to anomalous (non-Fickian) transport, indicating that both erosion and diffusion occurred during the dissolution process.

**Table IV:** *In Vitro* Release Kinetics of 3D Printed Tablets

		Zero-Order	First-Order	Hixson-Crowell	Ritger-Peppas	*n*
T 1	*k*	0.19801	0.46799	0.12868	0.37044	0.66972
	*R*^*2*^	0.93277	0.99605	0.9869	0.99885	
T 2	*k*	0.19557	0.44522	0.12281	0.35077	0.69403
	*R*^*2*^	0.94044	0.99645	0.99138	0.99981	
T 3	*k*	0.23977	0.56133	0.15607	0.41175	0.69147
	*R*^*2*^	0.92928	0.99727	0.99217	0.99953	
T 4	*k*	0.29208	0.62336	0.17502	0.43679	0.74744
	*R*^*2*^	0.94256	0.99541	0.99716	0.99985	
T 5	*k*	0.36098	0.6872	0.19429	0.47292	0.71049
	*R*^*2*^	0.95756	0.99432	0.99429	0.99904	

According to these release kinetic equations, first-order and Hixson-Crowell equations each require only one parameter to be determined. At the same time, the Ritger-Peppas equation requires the determination of "*k*" and the exponent "*n*". Therefore, first-order and Hixson-Crowell equations were used to do correlations. Linear and quadratic regression analyses were conducted between the surface area of the tablets and the "*k*" values for the five tablet shapes. Table V shows a correlation between the "*k*" and "*b*" values, with *R*^*2*^ values exceeding 0.8 for both linear and quadratic regressions. The quadratic regression exhibited a stronger correlation than the linear regression. Therefore, this method can establish a correlation between dissolution curves, the tablets’ surface area, and "*b*". With a given "*b*" value, the dissolution curve, dissolution rate, and time for complete drug release can be predicted. Conversely, tablet shape can also be predicted and constructed with a given dissolution curve and expected dissolution rate over time.

**Table V:** Correlations of the dissolution curves and parameters of the printed tablet

	First-order	Hixson-Crowell
Linear	*k* = *0.0027A(b)—0.3975*	*k* = *0.0008 A(b)—0.1291*
*R*^*2*^	0.8353	0.8436
Quadratic	*k* = *-4* × *10*^*−5*^*A(b)*^*2*^ + *0.0332 A(b)—5.9321*	*k* = *-1* × *10*^*–5*^* A(b)*^*2*^ + *0.0097 A(b)—1.7348*
*R*^*2*^	0.9148	0.9198

Where *A(b)* is the Eq. (9).

## Conclusion

In this study, we applied mathematical expressions to describe object surfaces and successfully constructed a series of tablet geometries precisely. These tablets were produced using thermal melt extrusion and fused deposition modeling 3D printing technologies. We established relationships between curve fitting and tablet structural parameters through a series of assessments. This approach aims to facilitate diverse and precise designs of 3D printed tablet structures, enabling their application in a broader range of scenarios. The ability to predict dissolution curves based on tablet structure, and vice versa, holds promising prospects for tablet design and the application of 3D printing in pharmaceuticals. This approach caters to personalized and tailored production needs, meeting diverse pharmaceutical manufacturing demands.

## Data Availability

All data generated or analysed during the study are included in this article.
